# Transcriptomic analysis of LMH cells in response to the overexpression of a protein of *Eimeria tenella* encoded by the locus ETH_00028350

**DOI:** 10.3389/fvets.2022.1053701

**Published:** 2022-11-21

**Authors:** Xiao-Jing Wu, Jin Gao, Bing-Jin Mu, Lin-Mei Yu, Zi-Rui Wang, Wen-Bin Zheng, Wen-Wei Gao, Xing-Quan Zhu, Qing Liu

**Affiliations:** ^1^Laboratory of Parasitic Diseases, College of Veterinary Medicine, Shanxi Agricultural University, Taigu, Shanxi, China; ^2^Key Laboratory of Veterinary Public Health of Higher Education of Yunnan Province, College of Veterinary Medicine, Yunnan Agricultural University, Kunming, Yunnan, China

**Keywords:** *Eimeria tenella*, LMH cells, dense granule protein 9, overexpression, transcriptome

## Abstract

A protein of *Eimeria tenella* (encoded by the locus ETH_00028350) homologous to *Toxoplasma gondii* dense granule protein 9, designated as EtHGRA9 hereafter, was reported to be expressed in all life cycle stages of *E. tenella*. However, no data are currently available regarding its functional properties. In the present study, a recombinant vector harboring a 741 bp gene segment encoding the mature form of EtHGRA9 was constructed and transfected into leghorn male hepatoma (LMH) cells. Then, transcriptomic analysis of the transfected LMH cells was carried out by using a high-throughput RNA-seq technology. The LMH cells overexpressing EtHGRA9 was validated by means of Western blotting as well as indirect immunofluorescence staining. The results demonstrated that the expression of 547 genes (275 upregulated genes and 272 downregulated genes) was altered by EtHGRA9. The quantitative real-time polymerase chain reaction (qRT-PCR) validation of the ten genes with differential expression between the two groups was consistent with the transcriptome analysis. According to pathway enrichment analysis for the obtained differentially expressed genes, seven pathways were significantly affected by EtHGRA9, such as cytokine-cytokine receptor interaction, MAPK signaling pathway, and protein processing in endoplasmic reticulum. Our data reveal several possible roles of EtHGRA9 in immune or inflammatory responses, which paves the way for a better understanding of the molecular interplay between *E. tenella* and its host.

## Introduction

*Eimeria* species are obligate intracellular apicomplexan protists that have been found in poultry, rabbits and other animals (1, 2). Coccidiosis is considered as an economically important disease of the poultry industry, and the total cost of coccidiosis in chickens was approximately £10.36 billion worldwide in 2016 (3). Seven *Eimeria* species have been reported to be associated with chicken coccidiosis (4). *Eimeria tenella*, one of the most virulent pathogens of coccidiosis, has been widely studied (5, 6).

Apicomplexan parasites such as *Toxoplasma gondii* harbor three sets of specialized secretory organelles, namely dense granules, micronemes and rhoptries (7, 8). A large number of proteins derived from these secretory organelles of *T. gondii* contribute to parasite invasion and survival, such as microneme protein 1 and dense granule protein 15 (9–11). Several proteins identified in *E. tenella* share homology with *T. gondii* dense granule proteins (12). To date, however, there is limited information regarding the functional properties of those proteins. Of interest is a protein (encoded by the locus ETH_00028350) homologous to *T. gondii* dense granule protein 9, hereafter designated as EtHGRA9, which was reported to be expressed in all *E. tenella* stages (13).

The leghorn male hepatoma (LMH) cell line with an epithelial phenotype is often used for studying host-pathogen interactions in the gastrointestinal tract of poultry (14). Furthermore, *E. tenella* resides inside the chicken intestinal epithelial cells (15). Hence, this study utilized the LMH chicken epithelial cell line as an *in vitro* model to investigate the biological roles of EtHGRA9 in the interplay between *E. tenella* and its host. To achieve this goal, the segment of EtHGRA9 gene was amplified by PCR from complementary DNA (cDNA) of *E. tenella* sporulated oocysts, and then a recombinant vector harboring this gene fragment was constructed. Thereafter, transcriptional changes in the LMH cells transfected with EtHGRA9 overexpression plasmid or empty vector were examined.

## Materials and methods

### Parasites and cell line

Total RNA was extracted from *E. tenella* SD-01 strain, followed by cDNA generation. The LMH cells were grown under the normal culture condition (5% CO_2_, 37°C) in Dulbecco's modified eagle medium (DMEM) containing 10% fetal bovine serum (FBS), 100 μg/mL streptomycin, and 100U/mL penicillin (16).

### Construction of the recombinant plasmid

**Figure 1:**
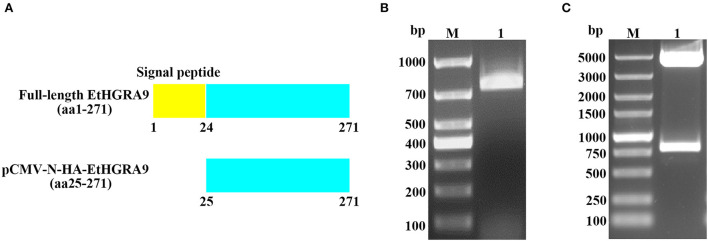
Construction of the pCMV-N-HA-EtHGRA9 expression vector. **(A)** The full-length EtHGRA9 protein and the region used for construction of the recombinant plasmid. **(B)** The EtHGRA9 gene segment amplified by PCR. **(C)** The constructed pCMV-N-HA-EtHGRA9 plasmid was confirmed by restriction enzyme digestion.

The gene fragment (Figure 1A) was amplified from the produced cDNA by using two gene-specific primers designed based on the mRNA sequence of EtHGRA9 published in GenBank (XM_013377103). The primer sequences are as follows: EtHGRA9-F (5′-GGATCCGATTCAAGCGAACGAAGGAG-3′) and EtHGRA9-R (5′-CCGGAATTCTTAGTTCAGCACATCCAGTT-3′). The PCR products were purified and subjected to digestion with restriction enzymes EcoRI and BamHI, followed by DNA ligation to construct the pCMV-N-HA-EtHGRA9 expression vector. After transformation into *Trans*5α chemically competent cells (TransGen Biotech, Beijing, China), the PhasePrep EndoFree Maxi Kit (Aidlab, Beijing, China) was used for isolation of plasmid DNA according to the manufacturer's specifications. Validation of the recombinant plasmid was performed by means of restriction digest analysis and sanger sequencing (Sangon Biotech, Shanghai, China).

### Transfection of LMH cells

Transfection of LMH cells was conducted by using the Xfect™ Transfection Reagent (Takara, Dalian, China) following the manufacturer's specifications. For indirect immunofluorescence assay, 0.75 μg empty control vector and overexpression plasmid were diluted separately in 25 μL transfection buffer, followed by mixing with 0.225 μL Xfect polymer to allow Xfect-DNA nanoparticles to form. The nanoparticle complex solution dropwise was added to the supernatant of the cultured cells with gentle shaking. The culture medium containing nanoparticle complexes was removed after 4 h, and 500 μL DMEM supplemented with 10% FBS was added.

With regards to Western blotting and transcriptome sequencing analysis, the cells were seeded into T25 flasks. For plasmid transfection, 12.5 μg plasmid DNA was diluted to a final volume of 100 μL. Additionally, 3.75 μL Xfect polymer was used.

### Indirect immunofluorescence assay

On day 2 post-transfection, the transfected LMH cells were fixed with 2% paraformaldehyde for 10 min, followed by treatment with 0.1% Triton X-100. Then, the cells were blocked with 5% bovine serum albumin. Following washing, the cells were incubated with mouse anti-HA tag antibody (Invitrogen, Carlsbad, CA, USA) for 1 h. Then, the cells were washed three times with PBS, followed by incubation with FITC-conjugated goat anti-mouse IgG (Abcam, Cambridge, UK) at 37°C for 30 min. An inverted fluorescence microscope (Nikon, Tokyo, Japan) was used to examine the fluorescent signal.

### Western blotting

For Western blotting analysis, the transfected LMH cells were lysed by using RIPA lysis buffer (Beyotime, Nantong, China) on day 2 post-transfection. The protein samples were separated by sodium dodecyl sulfate-polyacrylamide gel electrophoresis. Then, the separated proteins were transblotted onto a polyvinylidene difluoride membrane (Millipore, Bedford, MA, USA). Subsequently, the membrane was blocked with 5% skim milk in Tris-buffered saline with Tween-20 (TBST, 50 mM Tris, 150 mM NaCl, 0.05% Tween 20, pH 7.4) for 1 h at room temperature, and then incubated with the primary antibody (mouse monoclonal antibody to HA tag). Afterwards, the membrane was rinsed with TBST, followed by incubation with the secondary antibody (goat anti-mouse IgG-HRP) for 1 h. The immunosignal was examined using the ECL reagent (Thermo Scientific, Waltham, MA, USA).

### Transcriptome sequencing and bioinformatics analysis

The transfected cells were collected and submitted for RNA-seq, and each sample included three biological replicates. Total RNA was extracted from the transfected cells by using TRIzol Reagent, and RNA quality was evaluated using Agilent 2100 Bioanalyzer (Agilent Technologies, Palo Alto, CA, USA). Messenger RNA (mRNA) was purified from the extracted total RNA using poly-T oligo-attached magnetic beads. Then, the mRNA was split into fragments using divalent cations under elevated temperature and used for cDNA generation. Following construction of transcriptomic libraries, the paired-end sequencing was carried out by Novogene Corporation (Beijing, China). After removing low-quality reads from raw data and reads containing adapter or ploy-N, high-quality clean reads were mapped against the chicken (*Gallus gallus*) reference genome (16).

The “DEseq2” package in R (v1.20.0) was used for identification of differentially expressed genes (DEGs) between the two groups (17). A *P*-value less than 0.05 and a |log_2_ fold change|>1.0 were defined as the threshold of significance for differential expression. The clusterProfiler software package of R software (v3.8.1) was used to perform Gene Ontology (GO) functional annotation and Kyoto Encyclopedia of Genes and Genomes (KEGG) analysis (18). GO terms or pathways were considered to be significantly enriched if *P-*value < 0.05.

### Validation of RNA-seq data by qRT-PCR

Quantitative real-time polymerase chain reaction (qRT-PCR**)** was carried out to verify the results of transcriptome analysis. Briefly, GAPDH was selected as an internal standard for normalizing gene expression, and three reactions were performed per biological replicate. Ten DEGs were randomly selected for validation, and the control group genes were used as the reference. Details of the primers used to verify the sequencing data are shown in Table 1. The amplification protocol of qRT-PCR was conducted as follows: 95°C for 30 s, 40 cycles of 95°C for 15 s, 60°C for 30 s. Relative expression of mRNA was calculated using the 2^−ΔΔCT^ method (19).

**Table 1:** Primer sequences used in the present study.

**Gene ID**	**Gene name**	**Forward primer (5^′^-3^′^)**	**Reverse primer (5^′^-3^′^)**
770592	MT3	ACTCCCAGGACTGCCCTTGT	CAGCAGGAGCAGCAGCCTTT
421589	FNDC1	GGTTTCTTTGCCATCACGGAGT	GCTTGTAATCCCACCTTGCTGA
423931	BAG3	CGATACAAAGCAGAAGCCGTG	AGATTGAGGCTCAGGTCCGTGT
396491	LGALS1A	ATAATCGCACCGAATGCCAAA	CCACTCTTCCATTTTCTTTGAGTTG
428310	HSPB9	ACGCAGAACACGGACGAGAA	TTTGCTGACAGCTCCATCCTT
772158	LOC772158	TCCTACAAGTACGAGGTGCTGAAG	CATCACGCCTTGGCTCTCTC
374211	CRABP1	GCAAACAAACTCTTATTGAGGGAG	ACAACATCATCAGCACCAAAGG
396159	ST6GALNAC2	GTTTCTGCTTCTTGCTCCTCG	CTCCTGTTGGTGGCTGTTGTCT
100857679	CORO6	ACATCCGCGTCTCCAAGGTG	CGATGACGTTGTCGTTGTGG
100857703	CORF2	AGGGAAGGTGACGAAGGGT	ATCTCATGGGAGAGGCGGTGT

## Results

### Confirmation of the overexpression plasmid

As shown in Figure 1B, the gene segment coding for the mature peptide of EtHGRA9 was successfully amplified by PCR. The EtHGRA9 overexpression plasmid was firstly validated by restriction digestion analysis (Figure 1C), and then confirmed by DNA sequence analysis (data not shown).

### Confirmation of LMH cells overexpressing EtHGRA9

**Figure 2:**
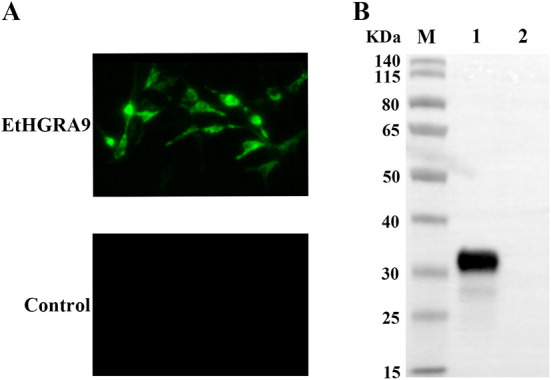
Expression analysis of EtHGRA9 in the transfected LMH cells. **(A)** Fluorescence microscopic photographs of the transfected LMH cells labeled by HA-tag mouse monoclonal antibody. **(B)** Western blot analysis of the transfected LMH cells using HA-tag mouse monoclonal antibody as the primary antibody. Lane 1: protein extracts from the LMH cells transfected with the EtHGRA9 overexpression plasmid; lane 2: protein extracts from the LMH cells transfected with the empty plasmid.

Expression of EtHGRA9 in the LMH cells transfected with pCMV-N-HA-EtHGRA9 was examined by using the indirect immunofluorescence test. The results showed that LMH cells transfected with this plasmid were fluorescently labeled with the monoclonal antibody to HA tag, whereas LMH cells transfected with the empty plasmid showed no staining (Figure 2A). Also, expression analysis of EtHGRA9 in the transfected LMH cells was conducted by using Western blotting. The results showed that LMH cells transfected with EtHGRA9 overexpression plasmid expressed a 31-KDa EtHGRA9-HA fusion protein (Figure 2B, lane 1), which was not observed in the control group (Figure 2B, lane 2).

### Comparison of gene expression changes

**Figure 3:**
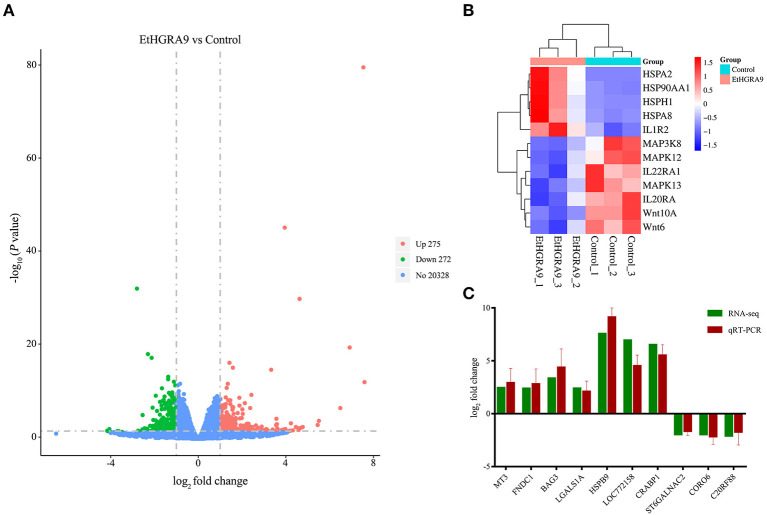
Differential expression analysis and qRT-PCR validation. **(A)** Volcano plot analysis of the DEGs between the LMH cells overexpressing EtHGRA9 and control cells. Green: downregulated genes; red: upregulated genes. **(B)** Heatmap of several DEGs. **(C)** Ten differently expressed transcripts were analyzed by qRT-PCR.

RNA samples showed high RNA integrity number (RIN) values (9.5 or greater), indicating the high quality of samples and no signs of degradation. Compared with the LMH cells transfected with the empty vector, 547 transcripts showed differential expression in the LMH cells overexpressing EtHGRA9. Of which, 275 transcripts were found to be upregulated, whereas 272 genes were significantly downregulated at the transcriptional level (Figure 3A, Supplementary Table 1). Several DEGs of interest are shown in Figure 3B. The mRNA expression levels of ten DEGs were measured to validate the RNA-seq results by the qRT-PCR method. As shown in Figure 3C, an agreement of the expression trend was observed.

### GO and KEGG analysis

**Figure 4:**
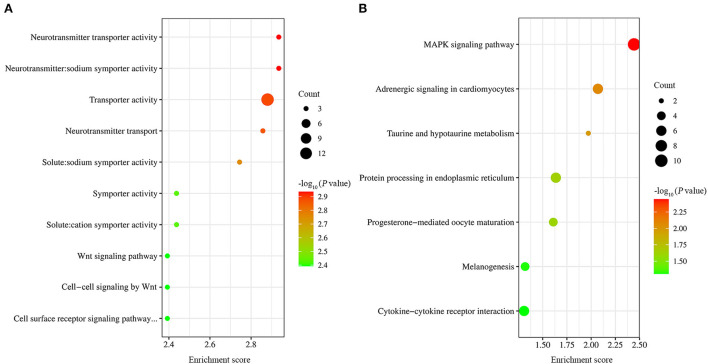
GO and KEGG analysis of DEGs. **(A)** The top ten GO terms with the most significant *P*-values. **(B)** The seven significantly enriched signaling pathway.

GO functional enrichment was carried out on the obtained differential genes. The results showed that a total of 54 GO terms were found to be significantly enriched, including 3, 26, and 25 GO terms for cell component, molecular function, and biological process, respectively (Supplementary Table 2). Of the top ten GO terms (Figure 4A), seven were neurotransmitter transporter activity, neurotransmitter: sodium symporter activity, solute:sodium symporter activity, transporter activity, neurotransmitter transport, symporter activity, and solute:cation symporter activity. The common DEGs classified into these GO terms included SLC6A4, SLC6A1 and SLC6A12. The rest of the ten GO terms were Wnt signaling pathway, cell surface receptor signaling pathway involved in cell-cell signaling, and cell-cell signaling by Wnt. The common DEGs classified into these GO terms included Wnt10A and Wnt6.

Also, KEGG pathway analysis of transcriptional data was conducted to investigate the cellular functions altered by EtHGRA9. The DEGs were significantly enriched in seven pathways, including MAPK signaling pathway, adrenergic signaling in cardiomyocytes, taurine and hypotaurine metabolism, protein processing in endoplasmic reticulum, progesterone-mediated oocyte maturation, melanogenesis, and cytokine-cytokine receptor interaction (Figure 4B). Details of the DEGs enriched in these pathways are shown in Supplementary Table 3.

## Discussion

*T. gondii* secretes dense granule proteins, which play a critical role in host-parasite interactions (20, 21). However, the functional properties of *E. tenella* dense granule proteins are still poorly understood. Herein, transcriptomic changes in LMH cells overexpressing EtHGRA9 were investigated by using RNA-seq approach. The LMH cells overexpressing EtHGRA9 were confirmed by western blotting and immunofluorescence analysis. All RNA samples exhibited high quality, with RIN values ranging from 9.5 to 10.0. Meanwhile, the reliability of the RNA-seq data was validated by qRT-PCR.

The top two significantly enriched terms were neurotransmitter transporter activity, and neurotransmitter: sodium symporter activity. Meanwhile, pathway analysis revealed that the DEGs were significantly enriched in protein processing in endoplasmic reticulum. The results were similar to that revealed by differential gene expression analysis in glioblastoma cells and normal human brain cells (22). A previous study suggested that the affected signaling pathway in cells infected with *E. tenella* included Wnt signaling pathway (23). Our results showed that the mRNA expression levels of Wnt10A and Wnt6 were altered in LMH cells overexpressing EtHGRA9, indicating that EtHGRA9 may be involved in regulating Wnt signaling pathway.

Protein processing in the endoplasmic reticulum pathway was reported to be involved in influencing protein folding in the endoplasmic reticulum (ER) (24), and excessive accumulation of unfolded proteins in the ER leads to ER stress (25). Previous studies showed that *T. gondii* infection could modulate host cell apoptosis through ER stress (26–28). Notably, ER stress is also associated with *E. tenella* infection (29). The DEGs enriched in protein processing in the endoplasmic reticulum pathway included HSPA2, HSPA8, HSP90AA1 and HSPH1, which are the immune-related genes (30). This indicated that EtHGRA9 may be involved in immune responses during *E. tenella* infection.

The DEGs enriched in MAPK signaling pathway included p38γ (MAPK12), p38δ (MAPK13) and MAP3K8. p38 MAPK family proteins can be divided into two subsets (p38α/p38β and p38γ/p38δ) (31). p38γ and p38δ was reported to be key components in innate immune responses (32). This indicated that *E. tenella* may impair innate immune responses by using EtHGRA9. MAP3K8 is an important regulator of pro-inflammatory cytokines (33, 34). Our results showed that compared with LMH chicken cells transfected with the empty plasmid, the expression of MAP3K8 in pCMV-N-HA-EtHGRA9-transfected cells was downregulated. This indicated that EtHGRA9 may possess anti-inflammatory properties. Intriguingly, *T. gondii* GRA9 was reported to be associated with inhibition of NLRP3 inflammasome activation and macrophage polarization (35).

Previous studies showed that cytokine-cytokine receptor interaction pathway was associated with *Eimeria* infection (29, 36). Of the DEGs enriched in this pathway, IL22RA1, IL1R2 and IL20RA are implicated in immune or inflammatory responses (37–39). This indicated that, besides affecting MAPK signaling pathway and protein processing in endoplasmic reticulum, EtHGRA9 may contribute to regulating immune or inflammatory responses through cytokine-cytokine receptor interaction pathway.

## Conclusion

In the present study, the roles of EtHGRA9 in the host-parasite interplay were investigated through transcriptomic analysis of the LMH cells overexpressing EtHGRA9. The results showed that the mRNA expression level of 547 genes in LMH cells was significantly altered by EtHGRA9, including 275 upregulated transcripts and 272 downregulated transcripts. Our transcriptomic data uncovered several potential roles of EtHGRA9 in immune or inflammatory responses.

## Data availability statement

The datasets presented in this study can be found in online repositories. The names of the repository/repositories and accession number(s) can be found at: https://www.ncbi.nlm.nih.gov/, PRJNA881463.

## Author contributions

QL and X-QZ conceived and designed the experiments. X-JW and JG performed the experiments, analyzed the data, and wrote the paper. B-JM, L-MY, Z-RW, and W-WG participated in the implementation of the study. W-BZ, QL, and X-QZ critically revised the manuscript. All authors have read and approved the final version of the manuscript.

## Funding

Project support was provided by the National Natural Science Foundation of China (Grant No. 31902298), the Fund for Shanxi “1331 Project” (Grant No. 20211331-13), the Science and Technology Innovation Program of Shanxi Agricultural University (2017YJ10), the Research Fund for Introduced High-level Leading Talents of Shanxi Province, the Special Research Fund of Shanxi Agricultural University for High-level Talents (Grant No. 2021XG001), and Yunnan Expert Workstation (Grant No. 202005AF150041).

## Conflict of interest

The authors declare that the research was conducted in the absence of any commercial or financial relationships that could be construed as a potential conflict of interest.

## Publisher's note

All claims expressed in this article are solely those of the authors and do not necessarily represent those of their affiliated organizations, or those of the publisher, the editors and the reviewers. Any product that may be evaluated in this article, or claim that may be made by its manufacturer, is not guaranteed or endorsed by the publisher.
